# The Role of Reactive Oxygen Species in Arsenic Toxicity

**DOI:** 10.3390/biom10020240

**Published:** 2020-02-05

**Authors:** Yuxin Hu, Jin Li, Bin Lou, Ruirui Wu, Gang Wang, Chunwei Lu, Huihui Wang, Jingbo Pi, Yuanyuan Xu

**Affiliations:** 1 Experimental Teaching Center, School of Public Health, China Medical University, No.77 Puhe Road, Shenyang North New Area, Shenyang 110122, China; yxhu@cmu.edu.cn (Y.H.); gwang@cmu.edu.cn (G.W.); cwlu@cmu.edu.cn (C.L.); 2 Program of Environmental Toxicology, School of Public Health, China Medical University, No.77 Puhe Road, Shenyang North New Area, Shenyang 110122, China; L1019Jin@163.com (J.L.); binlougg@gmail.com (B.L.); rui1224984970@163.com (R.W.); hhwang@cmu.edu.cn (H.W.); jbpi@cmu.edu.cn (J.P.)

**Keywords:** antioxidants, arsenic, Nrf2, ROS, signaling pathway

## Abstract

Arsenic poisoning is a global health problem. Chronic exposure to arsenic has been associated with the development of a wide range of diseases and health problems in humans. Arsenic exposure induces the generation of intracellular reactive oxygen species (ROS), which mediate multiple changes to cell behavior by altering signaling pathways and epigenetic modifications, or cause direct oxidative damage to molecules. Antioxidants with the potential to reduce ROS levels have been shown to ameliorate arsenic-induced lesions. However, emerging evidence suggests that constructive activation of antioxidative pathways and decreased ROS levels contribute to chronic arsenic toxicity in some cases. This review details the pathways involved in arsenic-induced redox imbalance, as well as current studies on prophylaxis and treatment strategies using antioxidants.

## 1. Introduction

Arsenic is the 33rd element in the periodic table of elements. It displays features of both a metal and a non-metal, and thus called metalloid. However, it is referred as a heavy metal from a toxicological point of view [1]. Arsenic can be found in soil, water, and air from natural and anthropogenic sources. Over time, arsenic has accumulated a variety of uses, for example in cosmetics, wood preservatives, cotton desiccants, pesticides, and even in the treatment of acute promyelocytic leukemia (APL) [2,3,4]. However, evidence of arsenic poisoning has occurred from oral ingestion of arsenic, when found as a contaminant in food or potable water and when used as a therapeutic. The major route of exposure is via drinking water due to natural contamination of groundwater by inorganic arsenic in the earth’s crust, which threatens the health of more than 140 million people worldwide [5,6]. Chronic exposure to arsenic has been associated with the development of a wide range of human cancers (e.g., lung, skin, liver, bladder, and kidney) [7], as well as other nonmalignant disorders (e.g., respiratory illnesses, cardiovascular diseases, diabetes, neurotoxicity, and renal diseases) [6,8,9,10]. 

Arsenic exists in nature as both an inorganic trivalent (arsenite: iAs III) and inorganic pentavalent forms (arsenate: iAs V), and is metabolized via biomethylation involving a two-electron reduction of pentavalent arsenicals followed by oxidative methylation to form organic pentavalent arsenicals [11,12]. Inorganic arsenic can be methylated to organics by S-adenosyl-L-methionine (SAM), including monomethylarsonic acid (MMA) and dimethylarsinic acid (DMA) in humans with trivalent (MMA III and DMA III, respectively) and pentavalent forms (MMA III and DMA III, respectively) [8]. Inorganic arsenicals are considered more toxic than organics [13]. This is substantiated by the observation that acute toxicity of inorganic arsenic is greater than organics, and methylated arsenicals are easier to excrete [14,15]. Therefore, arsenic methylation was once considered as a detoxification pathway. However, trivalent methylated arsenicals are found to be at least as cytotoxic as their parent compounds if not more so in several human cells [16,17]. MMA III and DMA III show a higher capacity to inhibit activities of many enzymes and are more genotoxic than iAs III [18,19]. Moreover, MMA III and DMA III are more potent inhibitors of glucose-stimulated insulin secretion (GSIS) in isolated islets than iAs III [20]. These studies obscure the role of arsenic methylation in its toxicity, and again suggest that variance in arsenic methylation contributes to inter-individual susceptibility to arsenic-induced adverse health effects [21,22,23].

Oxidative stress mediated by reactive oxygen species (ROS) is a common denominator in arsenic toxicity. Here, we summarized ROS-related pathways in arsenic toxicity, as well as prophylaxis and therapeutic potential of antioxidants to combat arsenic toxicity.

## 2. ROS and Arsenic Toxicity

**Figure 1:**
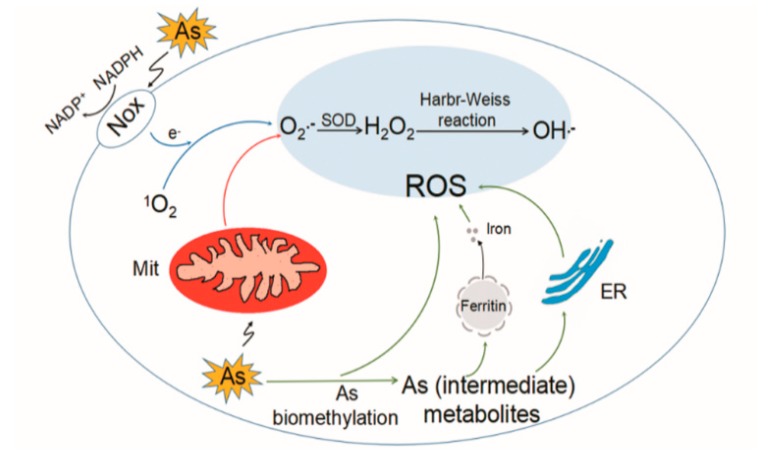
Mechanisms of generation of reactive oxygen species (ROS) induced by arsenic. Arsenic (As) induces significant ROS generation mainly through the mitochondrial (Mit) electron transport chain. Activation of nicotine adenine disphosphonucleotide (NADPH) oxidase (Nox) also contributes to the generation of superoxide anion (O_2_^−•^). Additionally, arsenic metabolism leads to the generation of ROS in cells. ^1^O_2_, singlet oxygen; H_2_O_2_, hydrogen peroxide; ^•^OH, hydroxyl radicals; ER, endoplasmic reticulum; SOD, superoxide dismutase.

The toxic mechanisms of arsenic are complex and not fully understood. At a biochemical level, iAs V can replace phosphate in several reactions. Arsenite (iAs III) and trivalent organic (methylated) arsenicals react with thiols (-SH) in proteins and inhibit their activity. Other mechanisms include epigenetic alteration, oxidative stress, inflammation, and autophagic defects [24,25,26]. ROS are formed in biological systems during the reduction of molecular oxygen and include the superoxide radical anion (O_2_^−•^), hydrogen peroxide (H_2_O_2_), hydroxyl radical (^•^OH), hydroperoxyl radical (HOO^•^), singlet oxygen (^1^O_2_), and peroxyl radical (ROO^•^) [27]. Arsenic induces formation of ^1^O_2_, O_2_^−•^, H_2_O_2_, ^•^OH, and ROO^•^. Formation of O_2_^−•^ and H_2_O_2_ in response to arsenic exposure in different cell lines are summarized by Shi et al. [28]. Mechanisms responsible for generation of ROS induced by arsenic are proposed as follows (Figure 1). (i) Mitochondria: Mitochondrial complexes I and III in electron transport chain are responsible for the production of O_2_^−•^. Arsenic shows mitochondrial toxicity by inhibiting succinic dehydrogenase activity and uncoupling oxidative phosphorylation with production of O_2_^−•^, which gives rise to other forms of ROS [29]. (ii) Nicotinamide adenine dinucleotide phosphate (NAD(P)H) oxidase (Nox): Nox is a membrane-associated enzyme involved in ROS generation in response to arsenic. The evidence mainly comes from endothelial cells and is reviewed by Ellinsworth [30]. (iii) Generation of ROS during formation of intermediate arsine species [31,32]. For example, dimethylarsenic peroxyl radical is formed during the metabolic processing of DMA [33]. (iv) Redox-active iron released from ferritin caused by methylated arsenic species [34]. (v) Under physiological conditions, the formation of ROS by arsenic lay on the oxidation of arsenite to arsenate [35]. (vi) Endoplasmic reticulum (ER): ER is suggested to be a source of ROS caused by DMA III [36]. (vii) Interference with cellular antioxidants, for example, superoxide dismutase (SOD), catalase (CAT) [37], glutathione (GSH), and GSH-related enzymes [38,39], which indirectly result in increased ROS levels. 

Despite of ROS overproduction reported in earlier studies applying high-dose arsenic in acute exposure, emerging evidence suggests that ROS do not increase when cells are exposed to an environmentally relevant dose of arsenic [40,41], especially after chronic exposure [42]. Treatment with 1 μM of arsenite for 4 h did not alter the amount of ROS in human bronchial epithelial cells (Beas-2B) [43]. Similarly, acute exposure to arsenite (2 or 4 h) below 10 μM of arsenite did not alter the intracellular amount of ROS in various cell lines, even with a more sensitive ROS detection method (electron paramagnetic resonance spectroscopy) [40,41]. Reduced intracellular ROS levels were observed in arsenic-transformed Beas-2B cells, and moreover were indicated as contributing to the acquisition of malignant phenotypes [42]. Our recent study found that arsenite-transformed human keratinocytes showed dysregulated autophagy with enhanced p62-NRF2 (nuclear factor (erythroid-derived 2)-like 2) feedback loop and decreased intracellular ROS levels [44]. In this scenario, constitutive Nrf2-mediated antioxidant response is frequently observed (see below). Thus, it is debated that adaptive antioxidant response is involved in toxicity caused by low-dose arsenic and especially during chronic exposure. Further understanding of the role of ROS in arsenic toxicity is needed. 

## 3. Involvement of ROS-Mediated Pathways in Arsenic Toxicity

Signal transduction pathways transmit extracellular signals from series intracellular signaling molecules into genes [45]. Arsenic can alter signal transduction via ROS alteration or reversible oxidation of -SH group in proteins, which leads to activation or inhibition of transcription factors and regulates gene transcription [46]. The major ROS-affected pathways in response to arsenic include Nrf2-antioxidant response element (ARE) signaling pathway, microRNAs (miRNAs), mitophagy pathway, tyrosine phosphorylation system, mitogen-activated protein kinases (MAPKs), nuclear factor κB (NF-κB), and activator protein-1 (AP-1) [47,48,49,50] (Table 1). 

### 3.1. Nrf2-ARE Pathway

Nrf2 is a master transcription factor in antioxidant system. Under physiological conditions, the protein level of Nrf2 is low because it binds to its negative regulatory factor kelch-like epichlorohydrin-associated protein 1 (Keap1), which forms the E3 ubiquitin ligase complex and facilitates ubiquitination and subsequent degradation of Nrf2 by the 26S proteasome [51]. When excessive ROS are generated, certain cysteine residues (C273, C288, and C151) in Keap1 sense the stress and are S-alkylated [52,53], leading to impairment of Keap1-mediated Nrf2 degradation [54]. Nrf2 then accumulates in the cytoplasm, translocates in the nucleus, dimerizes with small musculo-aponeurotic fibrosarcoma (Maf) proteins, and binds to the ARE motif in the promotor region of target genes, including various antioxidant enzymes and detoxification enzymes [55,56,57]. Therefore, the role of Nrf2-ARE pathway attracts much more attention in studies on arsenic toxicity. 

**Figure 2:**
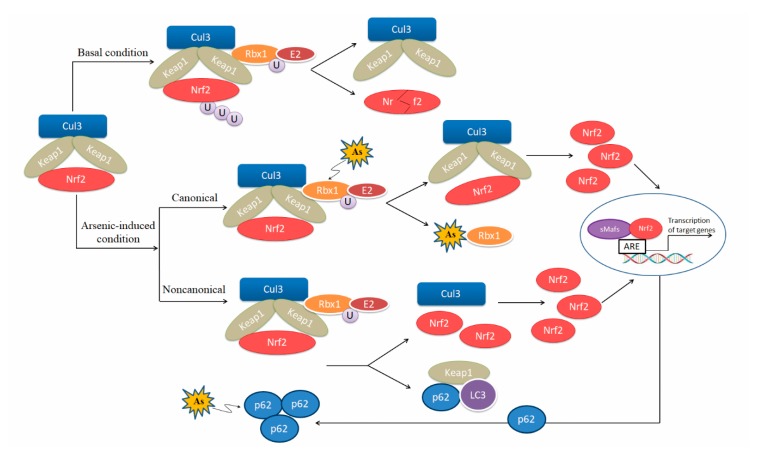
Regulatory models of the Nrf2-ARE pathway induced by arsenic. Under basal condition, Nrf2 is associated with Keap1 and degraded by proteasomes. Under arsenic-exposed condition, Nrf2 is activated via the canonical and noncanonical mechanisms. Arsenic binds to the Ring finger domain of RING-box 1 (Rbx1), which leads to the suppression of Cul3-Rbx1 E3 ubiquitin ligase activity, thereby activating the Nrf2-induced antioxidant signaling pathway via the canonical mechanism. Arsenic induces Nrf2 activation via the noncanonical mechanism by p62 accumulation due to dysregulated autophagy flux. *p62* is a downstream gene of Nrf2, forming a positive feedback loop with Nrf2.

Arsenic is an activator of Nrf2-Keap1 pathway [58,59,60,61,62]. Arsenite binds to the Ring finger domain of Ring-box 1 (Rbx1), which leads to the suppression of Cullin 3(Cul3)-Rbx1 E3 ubiquitin ligase activity, thereby activating the Nrf2-induced antioxidant signaling pathways [62]. Meanwhile, many research groups report that arsenic induces Nrf2 activation via the noncanonical mechanism, specifically by p62 accumulation due to dysregulated autophagy flux [43,63,64,65,66]. Accumulation of p62 results in sequestration of Keap1 in the autophagosomes and impairs Nrf2 degradation [67]. On the other hand, *p62* is a downstream gene of Nrf2, forming a positive feedback loop [63]. The loop may act as a critical molecular alteration in arsenic carcinogenesis [68]. In addition, arsenite is found to induce acetylation of Nrf2 by p300/CREB (cAMP response element binding protein) binding protein (CBP), which enhances Nrf2 binding capacity to promoter-specific DNA [69] (Figure 2). However, a significant down-regulation was found in cardiac *Nrf2* and *peroxisome proliferator-activated receptor-γ* (*PPAR-γ*) mRNA expression in arsenic-treated Sprague-Dawley (SD) rats compared with control rats [70]. Different response of Nrf2 to arsenic exposure is related to the strains and age of murines, as well as exposure time and dose of arsenic.

Nrf2 is considered as a protective factor against arsenic toxicity by reducing oxidative stress. Our previous study found that stable knockdown (KD) of *NRF2* in human keratinocytes (HaCaT) significantly increased the sensitivity to acute cytotoxicity of inorganic arsenite, whereas *KEAP1*-KD cells showed a significant resistance to arsenite toxicity [94]. When mouse macrophage cells (RAW 264.7) were exposed to arsenite, a marked increase in ROS occurred in *Nrf2*-KD cells compared to scramble cells [76]. However, abnormal activation of Nrf2 is suggested to be cancer-promoting [95,96]. On one hand, NRF2-dependent antioxidant and detoxification enzymes promote the detoxification and elimination of ROS to attenuate arsenic carcinogenesis. On the other hand, NRF2 activation may provide cell proliferation or survival advantage by mediating metabolic reprogramming [56,97] and contributing to apoptotic resistance [98,99,100,101], which are important events in the process of arsenic-induced malignant transformation. Long-term exposure to an environmentally relevant dose of arsenic may induce constitutive activation of Nrf2, leading to adaptive antioxidant response, and subsequently contributing to malignant transformation [60,74,102,103]. In chronic arsenic-exposed Chang human hepatocytes, protein levels of nuclear Nrf2 peaked at 8 weeks and significantly elevated afterwards, whereas cytosol Nrf2 did not show significant change, which suggests that chronic arsenic exposure may constitutively activate NRF2 by post-transcriptional mechanism [60]. Furthermore, downstream genes of NRF2; NAD(P)H dehydrogenase [quinone] 1 (*NQO1*); aldo-keto reductase family 1, member C2 (*AKR1C2*); and aldo-keto reductase family 1, member C3 (*AKR1C3*) were overexpressed in chronic arsenic-exposed HaCaT cells [102]. Our recent studies found that silencing *NRF2* in HaCaT cells abolished arsenic-induced acquisition of invasion capacity [44]. These data provide direct proof for the oncogenic role of Nrf2 in arsenic carcinogenesis. Taken together, Nrf2 pathway may exert dual roles in arsenic toxicity depending on the dose, exposure time, and cell types. Thus, concerns about strategy of using natural compounds, such as daphnetin (Daph) as an Nrf2 activator, for arsenic detoxification have been raised [104].

### 3.2. microRNAs

Epigenetic modifications contribute to toxic effects by arsenic exposure [105]. Alteration in miRNAs is one of these modifications and is closely related with intracellular ROS levels. He et al. found that chronic arsenic exposure lead to an overproduction of ROS, which induced activation of the miR-199a-5p/hypoxia inducible factor-1α (HIF-1α)/cyclooxygenase-2 (COX-2) pathway [77]. ROS inhibited miR-199a expression through increasing the promoter methylation of the *miR-199a* gene by DNA methyltransferase 1 [106]. miR-214 expression was transcriptionally repressed by Nrf2 through ARE within its promoter region in response to arsenic exposure in erythroid cells, and this repression was ROS dependent [78]. Not all alterations in miRNAs in arsenic-induced malignant transformation are related with ROS. Chen et al. found that Nrf2 was modulated by miR-155 in the process of malignant transformation induced by arsenite in human bronchial epithelial cells (16-HBE). However, there was no significant alteration in ROS production as determined by dichlorodihydrofluorescein diacetate (DCFH-DA) probe in the arsenic-transformed cells [107]. 

The levels of ROS can be regulated by miRNAs, as found in diseases such as cerebral ischemia, ischemia/reperfusion injury, and spinal cord injury. Overexpression of miRNA-20b increased the levels of adenosine 5’ triphosphate (ATP) and ROS in the cerebral ischemia of SD rats, whereas suppression of miRNA-20b decreased the levels of ROS [108]. Overexpression of miR-451 decreased apoptosis rate, ROS levels, and cleaved caspase-3 expression in the oxygen and glucose deprivation/reoxygenation cells [109]. *Lycium barbarum* polysaccharides (LBPs) reduced levels of ROS and nitric oxide (NO) induced by H_2_O_2_ through down-regulating miR-194 in PC-12 cells [110].

Understanding the molecular mechanisms of arsenic-induced toxicity, such as dynamics of ROS generation, miRNA expression, and the relationship between ROS and miRNAs, will certainly shed new light for future strategies against arsenic toxicity. However, so far, there are only a few studies exploring the molecular mechanism of arsenic toxicity in this regard. More studies are preferably needed to understand the potential relationship between ROS alteration and miRNA expression in response to arsenic exposure, of which research on the role of ROS in epigenetic dysregulation would be top priority.

### 3.3. Mitophagy

The mitochondrion is the major site for ROS production and leakage [111,112]. Moderate ROS levels are essential for cell proliferation and survival by mitophagy [113,114]. Excessive levels of ROS induce apoptotic signaling pathways. Furthermore, unceasing generated ROS in mitochondria lead to autophagy, apoptosis, or necrosis. On one hand, arsenic has mitochondrial toxicity, resulting in mitochondrial swelling and crista fragmentation, disturbing respiratory complex, and giving rise to ROS [115]. On the other hand, excessive ROS generation causes mitochondrial dysfunction [47,49]. 

Mitophagy is a type of autophagy physiologically responsible for mitochondrial quality control and mitochondrial ROS balance by removing damaged mitochondria [116,117,118,119]. Phosphate and tension homology deleted on chromsome ten (PTEN)-induced putative kinase 1 (PINK1)/Parkin is considered as one of the classical pathways required for mitophagy induction [119,120]. PINK1/Parkin-mediated mitophagy plays a protective role in some diseases [113,121]. For example, PINK1/Parkin-mediated mitophagy prevented renal tubular epithelial cells (RTEC) from apoptosis and tissue damage in contrast-induced acute kidney injury (CI-AKI) through reducing mitochondrial ROS and subsequent nucleotide-binding oligomerization domain-like receptors (NLR) protein 3 containing pyrin domain (NLRP3) inflammasome activation [113]. Increased mitochondrial fission promoted the survival of hepatocellular carcinoma (HCC) cells by facilitating autophagy and inhibiting mitochondria-dependent apoptosis, which was mediated via elevated ROS production [121]. 

Arsenic inhibits complex I in the mitochondrial electron transport chain, which leads to excessive generation of ROS, giving rise to lipid peroxidation and protein damage and the subsequent formation of mitochondrial permeability transition (MPT) [122]. Arsenite induces mitophagy via mitochondrial ROS and MPT [80]. PINK1/Parkin is activated upon mitochondrial membrane depolarization, a signal of mitochondrial dysfunction that results from multiple causes including hypoxia and impaired electron transport [123]. In arsenic toxicity, mitophagy exerts a dual role, facilitating cell survival either by eliminating damaged mitochondria or causing cell death. Arsenic-induced apoptosis in the pancreas of rats and insulinoma β-cell (INS-1) through impairment of mitophagy mediated by the ROS/Pparγ/PINK1/Parkin pathway [79]. Mitochondrial B cell lymphoma/leukemia-2 (Bcl-2)/adenovirus E1B 19 kDa-interacting protein 3 (BNIP3), a pro-apoptotic Bcl-2 homology 3 (BH3)-only protein, was activated as an upstream signal to increase the expression of caspase 3 and sequestosome 1 (SQSTM1), and contributed to increased cell death caused by arsenic [124]. BNIP3 also interacted with LC3 to target the damaged mitochondria and initiate mitophagy [125].

### 3.4. Alternative Pathways

#### 3.4.1. Tyrosine Phosphorylation

Tyrosine phosphorylation is an important posttranslational modification that is known to regulate receptor kinase (RK)-mediated signaling in mammals [126,127]. Auto-phosphorylation of specific tyrosine residues increases catalytic efficiency of the RK itself, whereas phosphorylation of additional tyrosine residues creates docking sites for downstream signaling molecules [128,129]. The tyrosine phosphorylation system is mediated by two important classes, receptor tyrosine kinases (RTKs) and nonreceptor tyrosine kinases (NTKs). The former includes growth receptors such as epidermal growth factor receptor (EGFR), platelet-derived growth factor receptor (PDGFR), and vascular endothelial grow factor (VEGF). The latter includes Src-family protein kinases. Activation of EGFR leads to activation of MAPK pathways. Arsenic exposure increases tyrosine phosphorylation in numerous proteins, with phosphorylation of EGFR as a central target [81,130,131]. The proposed mechanisms involve interaction of -SH groups on EGFR with arsenic and structural changes or dimerization of EGFR caused by ROS [81,130,131,132]. In addition, ROS may inactivate negative regulators of EGFR (namely protein tyrosine phosphatases, PTPs) via oxidation of cysteine residues in the active sites of these enzymes [133,134]. Following EGFR activation, Shc is recruited and phosphorylated at its tyrosine residues, which leads to enhanced Shc-Grb2 interaction and downstream signaling transduction including MAPKs [81,133]. In vivo and in vitro studies also found that arsenic induced Src activation in various cell lines [83,84,135], which is proposed to be mediated by direct interactions between arsenic and vicinal -SH groups of Src [135]. ROS may participate in arsenic induced Src activation, but the evidence remains unclear. In addition, Src acts as the upstream of EGFR and MAPK signaling in response to arsenic exposure [135]. 

#### 3.4.2. MAPK Pathway

MAPKs regulate proliferation, gene expression, differentiation, mitosis, survival, and apoptosis in the cell, and can be activated by arsenic in a time- and dose-dependent manner when the concentrations range from 0.1 to 500 μM [136,137]. The mRNA levels of *p38*, *extracellular signal-regulated kinase* (*ERK*)1, *ERK2*, and *Jun N-terminal kinase* (*JNK*) and the protein levels of phosphorylated-JNK (p-JNK)/JNK were significantly elevated in cardiomyocytes by arsenic [137]. Arsenic damaged intestinal epithelial cells (HT-29) by increasing p38 phosphorylation [138]. It has been suggested that arsenic activates MAPK via EGFR-dependent signaling transduction, such as EGFR/MEK, EGFR/Ras/MEK, and Src/EGFR cascades [29,83,134,139,140,141,142,143], or EGF-independent signaling pathways, such as Shc [135]. Arsenic induced the expression of COX-2, VEGF, HIF-1, and interleukin- 6 (IL-6), which were regulated by ERK, JNK, and p38 MAPK in simian virus-40 (SV-40) immortalized human uroepithelial cells and mouse lymphatic endothelial cells, mediated by ROS [75,86,88]. In addition, arsenic differentially activated MAPK pathways to exert opposing toxic effects, which were time-, dose-, arsenic species-, and cell-type-dependent. For example, acute arsenic exposure activated ERK1/2 and JNK, whereas chronic arsenic exposure induced continuous p38 activation in human hepatocytes [60]. JNKs were activated and prevented from arsenic-induced malignant transformation through apoptosis induction in mouse epidermal JB6 cells [144]. On the contrary, JNKs were essential in regulating expression of mineral dust-induced gene (*Mdig*), and thereby mediated arsenic-induced malignant transformation of BEAS-2B cells [145]. 

#### 3.4.3. NF-κB Pathway

NF-κB is a transcription factor regulating cell-to-cell interaction, intracellular communication, cell recruitment, and transmigration. NF-κB activation induced by arsenic is dependent on the duration of exposure, dose of arsenic, and cell types. Arsenic ranging from 1 μM to 10 μM activated NF-κB [136,146], which is suggested to involve generation of ROS [147], and not depended on inhibitory protein κB (IκB) phosphorylation and degradation [148,149]; however, arsenic above 10 μM inhibited NF-κB [136,146] via Iκκ [150,151,152,153] or interference with DNA binding of NF-κB [146]. In addition, there is a dynamic interaction between Nrf2 and NF-κB pathways, which is complex and under elucidation [154]. Nrf2 is proposed to suppress production of cytokines driven by NF-κB [155]. *Nrf2*-deficient mouse astrocytes exhibited increased DNA-binding activity and overproduction of pro-inflammatory cytokines [156]. The overexpression of *Nrf2* decreased the level of p65 in inflammation reaction in human adenocarcinoma alveolar basal epithelial cells (A549 cell) [157]. p65 has a dual role in Nrf2 activation. In the cell where Nrf2 and NF-κB were simultaneously activated, p65 inhibited Nrf2-ARE-driven transcription activity by competing CREB binding protein (CBP) from Nrf2, and promoted recruitment of histone deacetylase 3 to ARE, leading to local histone hypoacetylation [158,159]. On the other hand, in acute myeloid leukemia cells, NF-κB subunits p50 and p65 induced transcription of Nrf2 by binding to κB sites in Nrf2 proximal promoter [160]. In human embryonic kidney (HEK) 293T cells, Ras-related C3 botulinum toxin substrate 1 (RAC1) induced NRF2 activation through NF-κB [161]. NF-κB signaling pathway disrupted PPARα/δ-mediated lipid homeostasis, which was NRF2-independent in arsenic-exposed BEAS-2B cells [90].

#### 3.4.4. AP-1 Pathway

AP-1 is a complex composed of homodimers or heterodimers of Jun and Fos proteins. It has a vital role in cell growth and apoptosis [162]. All MAPK cascades can induce AP-1 activation in response to arsenic [84,163,164,165]. Effects of arsenic on AP-1 are closely related to arsenic species, as well as time and concentration of exposure [163,166]. Trivalent methylated arsenicals were more potent than trivalent inorganic arsenic as inducers of c-Jun phosphorylation and AP-1 activation [163]. In cells transiently transfected with an AP-1-dependent promoter-reporter construct, methylated trivalent arsenicals methylarsine oxide (MAs III O) was more effective than iAs III in inducing the AP-1-dependent gene transcription [163]. Acute arsenic exposure increased AP-1 binding to DNA via *c-Jun* and *c-Fos*, whereas chronic exposure attenuated DNA-binding capacity of AP-1 [163,166,167]. c-Jun/AP-1 pathway-mediated cyclin D1 was indicated as one of the key events in cell malignant transformation caused by low-dose arsenic exposure [168]. 

#### 3.4.5. p53 Pathway

p53 is a well-known tumor suppressor and plays an importance role in DNA repair, cell cycle, and apoptosis. Reports on the role of arsenic exposure on p53 are controversial, varying with arsenic species, exposure time, and cell types. In a human lyphoblastoid cell line, arsenic induced p53 expression by an ataxia telangiectasia mutated (ATM) (a member of PI3-kinase-related protein kinase)-dependent pathway, which phosphorylated p53 at serine 15 [169,170]. In HaCaT cells, chronic arsenic exposure inactivated p53 via poly(ADP-ribosyl)ation [171]. However, HaCaT cells are immortalized with SV40, which are known to interfere with p53 expression. In human telomerase reverse transcriptase (hTERT)-immortalized human keratinocytes, exposure to low-dose arsenite inhibited p53 expression by transcriptionally upregulating murine double minute 2 (MDM2) or ERK2-mediated overexpression of MDM2 [172]. In arsenic-exposed MCF-7 cells, an S-phase cell cycle arrest was found to depend on activation of p53 downstream cellular defense enzymes (i.e., sestrin 1 (SESN1) and activating transcription factor 3 (ATF3)) that was triggered by ROS generation in the early stage [93]. Recently, it has been shown that *MDM2* is a downstream gene of Nrf2 and serves a link between Nrf2 and p53 in pancreas cancer [173]. Yet, involvement of Nrf2-MDM2-p53 pathway in arsenic-induced cancer is not clarified.

#### 3.4.6. Stress Granules (SGs) Pathway

The overproduction of ROS induced by arsenic exposure causes tremendously harmful outcomes to cells, organs, and organisms [174]. To protect against arsenic toxicity, cells quickly activate antioxidant systems. Stress granules (SGs), the non-membranous cytosolic structures consisting of mRNAs and proteins, are formed and have antioxidant activity during arsenic exposure [175,176,177]. After the formation of SGs, the elevation of ROS was suppressed and ROS-dependent apoptosis was inhibited [175]. Beyond their function in defencing arsenic toxicity, SGs have been proposed to alter multiple signaling pathways, such as the JNK, Wnt, and mammalian target of rapamycin (mTOR) pathways, by intercepting and sequestering signaling components [178]. Accordingly, SG formation is a marker of chemoresistance and is upregulated by the production of a prostaglandin (15d-PGJ2), which is controlled by NRF2, in mutant v-Ki-ras 2 Kirsten rat sarcoma viral oncogene homolog (KRAS) cells [179]. The two antioxidant systems mentioned above, NRF2 and SGs, may intertwine in response to environmental stress, although the underling mechanism is not fully understood. Further understanding of the role of ROS along with NRF2 and SGs in arsenic toxicity is needed.

#### 3.4.7. Metabolism Pathway

Metabolic reprograming is a feature of cancer cells, which usually show a strong dependence on aerobic glycolysis (the Warburg effect), increase in glutaminolysis, enhancement of macromolecule production and mitochondrial biogenesis, activation of the pentose phosphate pathway, and upregulation of amino acid and lipid metabolism [180]. Although metabolic reprograming has been implicated in carcinogenesis [181], few studies have been performed to investigate the role of ROS in arsenic-relevant cancer metabolism. The feature of metabolic reprograming in response to arsenic challenge is still not fully understood, albeit there is some evidence suggesting that arsenic induces overproduction of ROS and aerobic glycolysis. Chronic arsenite (75 ppb) exposure was shown to induce aerobic glycolysis while inhibiting mitochondrial oxidative phosphorylation in primary human cells and multiple cell lines (BEAS-2B, human prostate epithelial cell line (RWPE-1), human pulmonary epithelial carcinoma cell line (A549), primary human urothelial cells (HUC), and human dermal fibroblasts (HDF)) [182]. A similar phenomenon was also observed in *Caenorhabditis elegans* following 48 h arsenite exposure (50 to 500 μM) [183]. When human hepatocyte cells (HL-7702) were treated with different concentrations of arsenite (1 to 5 μM, 12 h), overproduction of ROS resulted from activated nicotine adenine disphosphonucleotide (NADPH) oxidase-mitochondria axis inactivated prolyl hydroxylases (PHDs), which led to protein accumulation of HIF-1α [184]. The latter is recognized as an inducer of aerobic glycolysis [182,185]. Reciprocal crosstalk between ROS and metabolism is vital to function and fate of cancer cells [186,187,188]. Thus, the mitochondria, a major source of ROS production and ATP synthesis, represent a potentially target for cancer therapy [188]. Some antioxidants (e.g., NADPH and GSH) and redox cofactors (e.g., Nicotinamide adenine diuncleotide hydrogen (NADH) and reduced Flavin adenine dinucleotide (FADH)) act as a bridge in redox regulation and metabolic reprograming [189,190,191]. ROS can consume reducing agents (NADPH and GSH) key for cell metabolism [192,193], and meanwhile activate Nrf2, AMPK, and HIF-1, which regulate metabolism and in turn fine tune ROS levels [194,195,196]. Therefore, the overproduction of ROS induced by arsenic is linked to metabolic reprogramming by direct or indirect ways. However, the exact order for the evolution of ROS and cell metabolism and their specific roles in arsenic carcinogenesis remain to be further investigated.

## 4. Potential Application of Antioxidants to Rescue Arsenic Toxicity

The first line of defense against acute arsenic toxicity is to reduce the amount of arsenic in the body [197]. The ideal arsenic-removal drug can interfere with the interactions of arsenic and molecules in the tissue. 2,3-dimercaptopropane-1-sulfonic acid (DMPS) and meso-2,3-dimercaptosuccinic acid (DMSA) are hydrophilic and belong to the mercapto family, which have vicinal dithiol moiety for the binding of metals [198,199]. These drugs or their analogs offer therapeutic benefit in acute arsenic poisoning when administered promptly [200]. Due to the limited ability of crossing the blood–brain barrier, loss of essential metals in the body, low cellular membrane penetration, and potential side effects in the kidney and liver, the use of metal chelators is limited [197,201].

**Figure 3:**
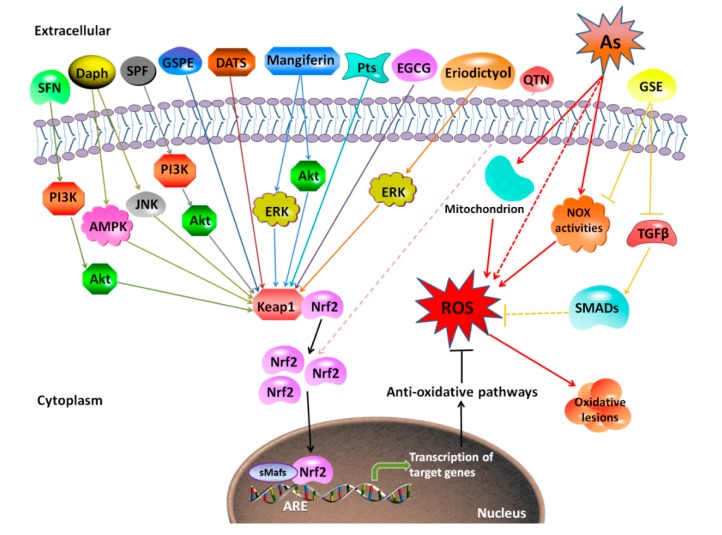
Mechanism of protective effects against arsenic-induced toxicity by antioxidants extracted from plants. The representative natural antioxidants extracted from plants show various mechanisms for protection against arsenic-induced toxicity. Most of them (SFN, Daph, SPF, GSPE, DATS, mangiferin, Pts, EGCG, and eriodictyol) promote the dissociation of Nrf2 with Keap1, promoting the expression of downstream genes of Nrf2, and finally increasing the antioxidant capacity. QTN is suggested to act though Nrf2 without clear evidence. Additionally, GSE protects against arsenic-induced oxidative damage through suppression of NOX-mediated ROS generation. SFN, sulforaphane; Daph, daphnetin; SPF, *Sorbus pohuashanensis* flavonoids; GSPE, grape seed proanthocyanidin extract; DATS, diallyl trisulfide; Pts, pterostilbene; EGCG, (-)-Epigallocatechin-3-gallate; GSE, grape seed extract; TGFβ, transforming growth factor-β; NOX, Nicotinamide adenine dinucleotide phosphate oxidase; SMADs, drosophila mothers against decapentaplegic protein.

Because it is well accepted that excessive generation of ROS plays an important role in the molecular mechanism of arsenic-induced toxicity and related diseases, application of antioxidants, especially extracts from plants, has been widely studied as therapeutics to counteract arsenic-induced toxicity. Some antioxidants involved in the methylation-mediated arsenic detoxification-excretion process, for example, GSH, can mitigate toxicity [202]. Some antioxidants decrease intracellular ROS levels via inhibiting mitochondrial respiratory chain complex I (e.g., metformin) [203]. Others present a protective role against arsenic-induced toxicity by regulating apoptosis-related molecular changes (e.g., diallyl trisulfide) [204,205]. Different types of antioxidants used for rescuing arsenic toxicity and their possible mechanisms are listed in Table 2. These antioxidants are classified as ROS scavengers, oxidative enzyme inhibitors, metal chelators, and antioxidant enzyme cofactors. The protective mechanisms of antioxidants extracted from the natural plants against arsenic-induced toxicity are shown in Figure 3. Besides reducing ROS, these antioxidants are involved in regulating signaling pathways such as Nrf2, NF-κB, MAPKs, transforming growth factor beta/Smad (TGF-β/Smad), and mammalian target of rapamycin/Akt (mTOR/Akt) [104,203,206,207,208,209,210,211].

Interestingly, different natural compounds used as antioxidants exert varied roles in Nrf2 activation in reducing arsenic toxicity. For example, tetramethylpyrazine (TMP) (50 μM or 100 μM) protected against arsenic-induced nephron toxicity by inhibiting Nrf2 activation, and accordingly reducing *Heme oxygenase-1* (*HO-1*) expression [209]. Pomegranate fruit extract (PFE) (0.2 mL of 0.2% of extract) reduced ROS generation in hepatocytes, thereby reducing arsenic-induced Nrf2 activation [207]. However, most antioxidants showed an effect on promoting Nrf2 activation in response to arsenic [26,70,104,211]. Dietary supplementation with SF (80 mg/kg BW) protected against arsenic-induced nephrotoxicity via the Phosphoinositide 3-kinase (PI3K)/Akt-mediated Nrf2 signaling pathway in the rat kidney [212]. Grape seed proanthocyanidin extract (GSPE) (10, 25, and 50 mg/L) activated Nrf2 signaling pathway to antagonize arsenic-induced oxidative damage, promoted arsenic methylation metabolism, and relieved arsenic-induced hepatotoxicity [26]. Alleviated arsenic toxicity in the liver and reproductive system by eriodictyol and lutein was via activating Nrf2 signaling pathway [213,214]. Co-treatment of antioxidant vitamins L-ascorbic acid and α-tocopherol attenuated toxicity induced by arsenic trioxide in H9c2 cardiomyocytes through activation of Nrf2 and Bcl2 transcription factors [215]. In contrast, PFE (0.2 mL of 0.2% of extract) reversed arsenic-induced hepatotoxicity with reduction in arsenic-induced Nrf2 activation [207], suggesting that other Nrf2-independent mechanisms are involved in attenuation of arsenic toxicity by this antioxidant.

Arsenic activates pro-inflammatory signal pathway and induces ROS generation, thereby resulting the activation of NF-κB [208]. TMP and PFE inhibited arsenic-induced NF-κB-inflammatory pathway [207,209]. In renal tubular epithelial cells, arsenic exposure leads to dynamic alterations of COX-2 expression, which was regulated by NF-κB. TMP and NAC pretreatment reversed COX-2 dynamic changes via suppression of NF-κB activation and β-catenin protein expression [208].

Some antioxidants attenuated arsenic-induced toxicity via MAPK activation. TMP inhibited arsenic-induced activation of MAPK family, and further reduced the expression of arsenic response protein 2 (ARS2), which contributed to its nephron protective effects [209]. As a prospective remedial agent, all-*trans* retinoic acid (ATRA) (0.5 mg/kg) reversed arsenic-induced oxidative stress and apoptosis by inhibiting the MAPK signaling pathways and repressing p53-dependent apoptosis in the rat uterus [206]. When hepatic cells were exposed to arsenic, p38 and JNK signaling pathways were activated. Carnosic acid (CA), which was commonly found in *Rosmarinus officinalis* and *Salvia officinalis*, significantly attenuated arsenic-induced phosphorylation of p38 and JNK [235].

TGF-β1 is a potent profibrogenic cytokine and can be initiated by oxidative stress [236]. Arsenic-induced rat renal injury was correlated with TGF-β1 induced fibrosis, as demonstrated by increased levels of TGF-β1 and pSmad2/3 in renal tissue. Grape seed extract (GSE) (100 mg/kg for male SD rats; 100 mg/L for rat hepatic stellate cells) reduced arsenic-stimulated oxidative stress, and thereby specifically suppressed TGF-β/Smad signaling [228]. (-)-Epigallocatechin-3-gallate (EGCG), eriodictyol, lutein, folic acid, zinc, and selenium were all able to elevate the expression of SOD, GSH, and CAT, and reduce the level of malondialdehyde (MDA) caused by arsenic, resulting in amelioration of arsenic-induced toxicity [214,220,232,233].

Generally speaking, accumulating evidence suggests that antioxidants have the potential to alleviate arsenic toxicity through reducing ROS generation, enhancing antioxidant capacity, regulating ROS-related signaling pathways, and keeping the balance of inflammation and immunomodulation. Natural antioxidants extracted from plants are more promising due to their rich sources, diversity, and few side effects. However, many studies on the role of plant extracts have not been systematically conducted. In vitro assessment results do not provide exact therapeutic implications because effectiveness plant extracts may be influenced by several physiopharmacological processes, such as absorption, distribution, metabolism, storage, and excretion, as well as bioavailability, and presence of co-antioxidants and ions [237]. Hence, a commonly suggested strategy, in which in vitro mechanic analysis goes after in vivo effect evaluation [238], is proposed to assess therapeutic potential of plant extracts in arsenic toxicity. In addition, there is a need to first identify the major target of arsenic-induced pathophysiological alterations. Many plant extracts have multiple functions in addition to severing as an antioxidant. Finally, plant extracts should be screened for low molecular antioxidants that are able to cross blood–brain barriers and reach other target organs. In addition to the above, more and more physiologically-based pharmacokinetic (PBPK) models have been used to predict the pharmacokinetic behavior of drugs in humans on the basis of preclinical data [239,240]. PBPK modeling, a good tool for evaluating and optimizing clinical trial design, provides an approach that enables the plasma concentration-time profiles to be predicted from in vitro and in vivo data [239,241,242], which can be used for choosing the dose of antioxidants.

## 5. Conclusions

An effectively preventive and therapeutic strategy for arsenic poisoning is still a challenge worldwide due to the incomplete understanding of underlying mechanisms. ROS alteration is a widely accepted key event in arsenic toxicity. Earlier studies with acute exposure and relatively high dose arsenic suggest that excessive levels of ROS disturb cellular signaling pathways, as well as damage macromolecules. The effects of antioxidants on arsenic toxicity are also assessed in a scenario of relatively high dose of arsenic. However, the mechanism and function of ROS may be different in the realistic environment, in which a relatively low-dose and long-term exposure to arsenic affects millions of people. In addition, in vitro assay concentrations of arsenic may misrepresent potential in vivo toxic effects and do not provide dose-response data that can be used for a risk assessment [243,244]; hence, the in vitro-to-in vivo extrapolation (IVIVE) by physiologically based toxicokinetic (PBTK) modeling was used to serve in vitro-in silico-based risk assessment [245]. It is of great importance to apply physiologically relevant doses during toxicological research. The role of ROS in arsenic toxicity should be fully clarified before the application of antioxidants. Of all the antioxidants investigated in arsenic toxicity, natural antioxidants extracted from plants are promising due to their rich sources, diversity, and few side effects, especially those with low molecular weight. More effective therapeutic value from plant extracts is expected on the basis of arsenic-induced pathophysiology targeting combined with in vitro and in vivo assessment.

## Figures and Tables

**Table 1:** Pathways related with ROS in arsenic toxicity.

Classification	Pathway	Model	Treatment	Key Findings	References
Nrf2-ARE (nuclear factor (erythroid-derived 2)-like 2- arsenic include Nrf2-antioxidant response element)	Nrf2/Heme oxygenase 1 (HO-1)/ROS	Human skin fibroblasts	0, 2.5, 5, or 10 μM As_2_O_3_ for 24 h	Arsenic exposure leads to upregulated expression of Nrf2 and its downstream target gene *HO-1* with reduced levels of ROS.	[71]
	Nrf2/HO-1, A170, Prx I	MC3T3-E1 cells	200-800 μM NaAsO_3_ or 50-100 μM NaAsO_2_ for 0, 4, 8, 12, 16, or 24 h	Arsenic induces Nrf2 activation, resulting in the transcriptional activation of *A170*, *HO-1*, and *Prx I* and accumulating high molecular mass forms of A170.	[58]
	H_2_O_2_/Nrf2/HO-1	JAR cells	5 µM As_2_O_3_ for 2 to 24 h; 0-10 µM As_2_O_3_ for 6 h	Arsenic exposure causes H_2_O_2_ over-production, and leads to increase of Nrf2/small Maf DNA binding activity and *HO-1* expression.	[72]
	Nrf2/HO-1, NAD(P)H dehydrogenase [quinone] 1 (NQO1)	MDA-MB-231 cells	0-50 µM NaAsO_2_ for 16 h; 0-2.5 µM monomethylarsonic acid (MMA) (III) for 16 h	Arsenic inhibits the activity of the kelch-like epichlorohydrin-associated protein 1 (Keap1)-Cul3 E3 ubiquitin ligase and then induces the Nrf2-dependent response.	[73]
	Nrf2/HO-1, NQO1, Glutamate-cysteine ligase catalytic subunit (GCLC)	HaCaT cells	100 µM NaAsO_2_ for 28 weeks	Arsenic-transformed cells show elevated expression of Nrf2 and its target genes, including *NQO1*, *HO-1*, and *GCLC*.	[74]
	ROS/Nrf2/HO-1	Mouse lymphatic endothelial cells	0, 5, or 7.5 μM As_2_O_3_ for 6 h	Arsenic causes ROS over-production and induces Nrf2 activation and *HO-1* expressions.	[75]
	Nrf2/NQO1, HO-1, GCLC/ROS/p38/ nuclear factor of activated T-cells c1 (NFATc1)	RAW 264.7 cells; mouse bone marrow-derived macrophages	5 ppm iAs in drinking water for 16 weeks; 0, 0.25, or 0.5 μM iAs for 7 days	Lack of Nrf2 increases arsenic-induced ROS levels and phosphorylation of p38, which aggravates the increase in osteoclastogenesis.	[76]
microRNAs	ROS/miR-199a-5p/HIF-1α/COX-2	BEAS-2B cells	1 μM NaAsO_2_ for 26 weeks, 0, 0.5, 1, or 2 μM NaAsO_2_ for 24 h	Arsenic-induced ROS inhibit miR-199a expression, and induce the expression of HIF-1α and COX-2.	[77]
	ROS/Nrf2/miR-214/AFT4	MEL cells	10 μM arsenic for 3 h	*miR-214* expression is transcriptionally repressed by Nrf2 in a ROS-dependent pathway, which leads to increased ATF4 protein content.	[78]
Mitophagy	ROS/Pparγ/phosphate and tension homology deleted on chromsome ten (PTEN)-induced putative kinase 1 (PINK1)/Parkin	INS-1 cells	0, 2, 4, or 8 mg/kg As_2_O_3_ (from gestation day 6 until pup postnatal day 42)	Arsenic induces ROS, which inhibits the expression of PPARγ and PINK1, and upregulates the expression of Bax.	[79]
	Mito O_2_^−•^, H_2_O_2_/ mitochondrial permeability transition (MPT)	U937, MCF-7, HT22, and NCTC-2544 cells	2.5 µM NaAsO_2_ for 16 or 48 h	Arsenite induces DNA damage via mitochondrial ROS and induction of mitochondrial permeability transition.	[80]
RTKs (receptor tyrosine kinases)/NTKs (nonreceptor tyrosine kinases)	ROS/epidermal growth factor receptor (EGFR), Shc/Ras/ERK	PC12 cells	400 μM NaAsO_2_ for 0, 10, 20, 30, 60 min	EGFR and Shc mediate in the activation of Ras/ERK signaling cascade by arsenite.	[81,82]
	ROS/c-Src/NF-κB	Porcine aortic endothelial cells	0 to 100 μM NaAsO_2_ for 24 h	H_2_O_2_ is sufficient for arsenite-induced stimulation of tyrosine kinases and activation of NF-κB.	[83]
	ROS/EGFR/MAP/ERK/AP-1	Mouse urinary bladder	0.002% or 0.01% NaAsO_2_ for 16 weeks	Arsenic-induced cell proliferation is correlated with the activation of MAP kinase pathway, leading to activation of ERK kinase and AP-1.	[82,84]
MAPKs (mitogen-activated protein kinases)	ROS/JNK, ERK/ gastrin-releasing peptide 78 (GRP 78), CHOP	Neuro-2a cells	0, 1, 3, 5, 7, or 10 μM As_2_O_3_ for 24 h	Arsenic induces ROS generation, causing cell death via both JNK/ERK-mediated mitochondria-dependent and GRP 78/CHOP-triggered apoptotic pathways.	[85]
	ROS/MAPK, PI3K/AKT/HIF-1a/COX-2, vascular endothelial grow factor (VEGF)	SV-HUC-1 cells	0, 1, 2, 4, 8, or 10 μM NaAsO_2_ for 24 h	Arsenic-induced COX-2, VEGF, and HIF-1 expression, mediated by ROS, is regulated by ERK, JNK, p38 MAPK, and PI3K/AKT.	[86]
	ROS/JNK, p38/ATF2	SV-HUC-1 cells	0, 1, 2, 4, 8, or 10 μM NaAsO_2_ for 24 h	Arsenic-induced ROS are involved in activation of JNK and p38 signaling pathways, which are responsible for ATF2 overexpression.	[87]
	ROS/ERK, JNK, and p38/COX-2	SV-HUC-1 cells	0, 1, 2, 4, 8, or 10 μM NaAsO_2_ for 24 h	Arsenic induces ROS, which result in an induction of COX-2 expression through activation of the ERK, JNK, and p38 MAPK pathways.	[88]
	ROS/ERK, JNK, and p38/IL-6, VEGF	SVEC4-10 cells	0, 5, or 7.5 μM As_2_O_3_ for 6 h	Arsenic causes ROS over-production and induces activation of ERK, JNK, and p38 MAPK, as well as expression of IL-6 and VEGF.	[75]
	ROS/ERK1/2/Beclin1, PINK1, Parkin 1, LCIIIB	Male Wistar rats	NaAsO_2_ (10 mg/kg) orally for 3 months	PKCδ is activated in the arsenic-intoxicated aged brains, which increases the expression of ERK1/2. ERK1/2 activates its downstream autophagic molecules Beclin1, PINK1, Parkin 1, and LCIIIB.	[89]
NF-κB (nuclear factor κB)	ROS/NF-κB/PPARα/δ	BEAS-2B cells	0, 2.5, 5, 10 or 25 μM As_2_O_3_ for 24 h; 2.5 μM As_2_O_3_ for 6 months	Arsenic induces ROS generation, enhancing NF-κB signaling and suppressing PPARα/δ signaling.	[90]
	ROS/c-Src/NF-κB	Porcine aortic endothelial cells	0 to 100 μM NaAsO_2_ for 24 h	H_2_O_2_ is sufficient for arsenite-induced stimulation of tyrosine kinases and activation of NF-κB.	[83]
AP-1 (activator protein-1)	ROS/ERK/AP-1/cyclin A	HaCaT and Int407 cells	0, 2 or 20 μM NaAsO_2_ for 24 h	Arsenic-induced cell proliferation is associated with enhanced ROS generation, ERK signaling, and cyclin A expression.	[91]
	ROS/AP-1	1RB3AN27 cells	0 to 10 μM NaAsO_2_ for 2 h or 72 h	Arsenic, in a dose-dependent manner, induces generation of ROS and activation of AP-1.	[92]
p53	p53/ROS/SESN1/ Cell division cycle 25A (CDC25A)	(MCF-7 (p53^+/+^)) or H1299 cells	5 μM NaAsO_2_ for 6, 12, 24, or 36 h	Arsenic activates p53-dependent transcription of ROS detoxification genes, which could be responsible for the S-phase cell cycle arrest.	[93]

**Table 2:** Antioxidants with prophylactic and therapeutic potential to rescue arsenic toxicity.

Classification	Compound	Model and Tissue	Treatment	Mechanism	References
ROS scavengers	Arjunolic acid	Female Wistar rats; serum	NaAsO_2_ (10 mg/kg for two estrous cycles); arjunolic acid (10 mg/kg for two estrous cycles)	malondialdehyde (MDA) ↓ conjugated diene (CD) ↓ ROS ↓ ovarian steroidogenesis ↑ Vitamin B_12_ ↑ folate↑	[216]
	Gallic acid (GA)	Male Wistar rats; heart and spleen	NaAsO_2_ (10 mg/kg for 21 days) GA (10 or 30 mg/kg for 7 days)	creatine kinase-MB (CK-MB) ↓ nitric oxide (NO) ↓ MDA ↓ glutathione (GSH) ↑ superoxide dismutase (SOD) ↑ glutathione peroxidase (GPx) ↑ catalase (CAT) ↑	[217]
	Grape seed proanthocyanidin extract (GSPE)	Human L-02 cells	NaAsO_2_ (25 μM for 24 h) GSPE (10, 25, or 50 mg/L for 24 h)	ROS ↓ MDA ↓ Nrf2 ↑ HO-1 ↑ NQO1 ↑ gultathione S transferases (GST) ↑	[26]
	Diallyl trisulfide (DATS)	Male albino rats; serum	Na_3_AsO_4_ (5 mg/kg for 28 days) DATS (20, 40, or 80 mg/kg for 28 days)	GSH ↑ SOD ↑ GPx ↑ CAT ↑ GST ↑ alkaline phosphatase (ALP) ↓ alanine aminotransferase (ALT) ↓aminotransferase (AST) ↓ ACP ↓	[204]
		Male albino rats; serum	Na_3_AsO_4_ (5 mg/kg for 28 days) DATS (20, 40, or 80 mg/kg for 28 days)	GSH ↑ SOD ↑ GPx ↑ CAT ↑ GST ↑ glutathione reductase (GR) ↑ glucose-6-phosphate dehydrogenase (G6PD) ↑ Total sulfhydryl groups (T-SH) ↑ vitamin C (VC) ↑ vitamin E (VE) ↑ Nrf2 ↑ HO-1 ↑ ROS ↓ NO ↓	[218]
	(-)-Epigallocatechin-3-gallate (EGCG)	Sprague-Dawley (SD) rats; liver	NaAsO_2_ (5 mg/kg/day for 30 days) EGCG (50 mg/kg/day for 30 days)	Nrf2 activation ↑	[210]
		Male BALB/c mice; serum, thymus, and spleen	NaAsO_2_ (10 mg/kg/day for 30 days) EGCG (10 mg/kg/day for 30 days)	ROS ↓ Caspase-3 activation ↓	[219]
		Swiss albino mice; serum and spermatozoa	Na_2_HAsO_4_·7H_2_O (200 ppm for 40 days) EGCG (20 mg/kg for 40 days)	ROS ↓ GSH ↑ CAT ↑ MDA ↓	[220]
	Tetramethylpyrazine (TMP)	Human kidney 2 (HK-2) cells	NaAsO_2_ (10 μM for 24 h) TMP (50 µM or 100 µM for 24 h)	ROS ↓ NF-κB ↓ COX-2 ↓ Mitochondrial dysfunction ↓ GSH ↑	[208]
		Human HK-2 cells	NaAsO_2_ (10 μM for 6 or 24 h) TMP (50 µM or 100 µM for 6 or 24 h)	Arsenic-induced MAPKs, AP-1, Nrf2, and NF-κB pathways ↓ HO-1 ↓ ARS2 ↓	[209]
	Flaxseed oil (FXO)	Male Wistar rats; kidney and blood	Na_2_HAsO_4_ (20 mg/kg for 4 days) FXO (15% by weight for 18 days)	Free radicals ↓ ROS ↓ Membrane organization and functions ↑	[221]
	Pomegranate fruit extract (PFE)	Male Swiss albino mice; liver	NaAsO_2_ (0.01, 0.05, or 0.1 mg/L for 30 days) PFE (0.2 mL of 0.2% of extract for 30 days)	ROS ↓ Nrf2 ↓ p53 ↓ miR-34a ↓ Apoptosis ↓	[207]
	Eriodictyol	Male Wistar rats; liver	As_2_O_3_ (3 mg/kg at day 1, 4, and 5) Eriodictyol (10, 20, or 40 mg/kg, 1 h before or after As_2_O_3_ treatment)	ROS ↓ MDA ↓ SOD ↑ GPx ↑ CAT ↑ Nrf2 ↑ HO-1 ↑	[214]
	Sulforaphane (SFN)	Male albino Wistar rats; renal	NaAsO_2_ (5 mg/kg for 28 days) SFN (80 mg/kg for 28 days)	B-cell lymphoma 2-associated X protein (Bax) ↓ ROS ↓ SOD ↑ CAT ↑ B-cell lymphoma 2 (Bcl2) ↑ Arsenic-induced nephrotoxicity ↓ PI3K/Akt ↑ Nrf2 ↑	[212]
	*Syzygium cumini* seed extract (SCE)	Wistar albino rats; blood and liver	NaAsO_2_ (100 ppm for 60 days) SCE (200, 400 mg/kg for 60 days)	AST ↓ ALP ↓ ALT ↓ SOD ↑ GSH ↑ CAT ↑	[222]
	Daphnetin (Daph)	Human Beas-2B cells	NaAsO_2_ (25 μM for 20 h) Daph (2.5, 5 or 10 μg/mL for 20 h)	Bax ↓ ROS ↓ Bcl2 ↑ Nrf2 ↑ HO-1 ↑	[104]
	Vitamin C or Vitamin E	Male albino rats; plasma, liver, and kidney	NaAsO_2_ (100 ppm for 30 days) Ascorbic acid (200 mg/kg for 30 days) α-tocopherol (400 mg/kg for 30 days)	Protein carbonyl content and DNA damage ↓	[223]
	Folic acid	Male albino rats; serum	As_2_O_3_ (3 mg/kg/day for 30 days) Folic acid (36 μg/kg/day for 30 days)	NO ↓ OH^-^ ↓ SOD ↑ GSH ↑ CAT ↑ MDA ↓	[224]
	Lutein (LU)	Male Kunming mice; plasma and testis	As_2_O_3_ (5 mg/kg/day for 5 weeks) Lutein (40 mg/kg/day for 5 weeks)	Nrf2 ↑ HO-1 ↑ NQO1 ↑ GST ↑ SOD ↑ GSH ↑ MDA ↓	[213]
	All-*trans* retinoic acid (ATRA)	SD rats Uteri and serum	NaAsO_2_ (4 ppm for 28 or 56 days) ATRA (0.5 mg/kg for 28 or 56 days)	MAPK signaling components ↓ p53-dependent apoptosis ↓	[206]
	Glutathione	Female albino mice; urine, liver	NaAsO_2_ (50 mg/L for 10 days) GSH (200, 400, or 800 mg/kg after NaAsO_2_ treatment)	Arsenic methylation ↑ Arsenic excretion ↑	[202]
	Melatonin	Male Wistar rats; liver	As_2_O_3_ (10 mg/mL for 4 days) Melatonin (5, 10, or 20 mg/kg for 8 days)	AST ↓ ALT ↓ MDA ↓ ROS ↓ SOD ↑ GPx ↑ CAT ↑ Nrf2 ↑ HO-1 ↑ p-Akt/Akt ↑ p-PI3K/PI3K ↑	[225]
Oxidative enzyme inhibitors	*Sorbus* *pohuashanensis* flavonoids (SPF)	BALB/c mice; heart H9c2 cells	As_2_O_3_ (1 mg/kg for 14 days) SPF (5, 10, or 20 mg/kg for 14 days)	creatine kinase (CK) ↓ CK-MB ↓ glutamic oxaloacetic transaminase (GOT) ↓ lactate dehydrogenase (LDH) ↓ ROS ↓ Caspase-3, -8, -9 ↓ SOD ↑ glutathione peroxidase (GSH-Px) ↑ CAT ↑ Bcl2/Bax ↑ Nrf2 ↑ HO-1 ↑ p-Akt/Akt ↑	[226]
	Pterostilbene (Pts)	HaCaT cells and JB6 cells	NaAsO_2_ (25 mM for 24 h) Pts (3.75, 7.5, 15, or 30 mM for 1 h)	Bax ↓ MDA ↓ ROS ↓ Caspase 3 ↓ Nrf2 ↑ HO-1 ↑ Bcl-2 ↑ SOD ↑	[227]
	Grape seed exact (GSE)	Male SD rats; liver	NaAsO_2_ (30 ppm for 12 months) GSE (100 mg/kg for 12 months)	p-Smad2/3 ↓ TGF-β ↓ Nox2 ↓ Nox4 ↓ p47phox ↓	[228]
		Rat hepatic stellate cells	NaAsO_2_ (2 μM for 24 h) GSE (100 mg/L for 20 min)	TGF-β/Smad signaling ↓ NADPH oxidase activities ↓ ROS ↓	
		Male SD rats; renal	NaAsO_2_ (30 ppm for 12 months) GSE (100 mg/kg for 12 months)	TGF-β/Smad signaling ↓ NADPH oxidase activities ↓ ROS ↓	[229]
	Metformin	Male Kunming mice; liver	As_2_O_3_ (6 μM for 48 h); Metformin (5 mM for 48 h) As_2_O_3_ (12 mg/kg/day for 3 days) Metformin (200 mg/kg/day for 3 days)	mitochondrial respiratory chain complex I ↓ NADH/NAD^+^ ↑ ROS ↓	[230]
		Intrahepatic cholangiocarcinoma cell lines (CCLP-1, RBE, and HCCC-9810)	As_2_O_3_ (3 μM for 24 or 72 h); metformin (10 mM for 24 or 72 h)	mTORC1 ↓ p38 MAPK ↓ ERK3 ↑	[203]
Metal chelators	Mangiferin	Male Swiss albino mice; lung	NaAsO_2_ (10 mg/kg for 3 months) Mangiferin (40 mg/kg for 5 weeks)	LDH ↓ MPO ↓ LPO ↓ ROS ↓ TNF-α ↓ Nrf2 ↑ HO-1 ↑ SOD ↑ GST ↑ GR ↑ GPx ↑ GSH ↑	[211]
		HepG2 cells	NaAsO_2_ (10 mg/kg for 28 days) Mangiferin (40 mg/kg for 5 weeks)	ALP ↓ ALT ↓ LDH ↓ ROS ↓ GSSG ↓ MDA ↓ SOD ↑ GST ↑ GR ↑ Catalase ↑ GSH ↑ GSH/GSSG ↑	[231]
	3,5,7,3′,4′-Pentahydroxy flavone (QTN)	Male SD rats; serum	NaAsO_2_ (5 mL/kg for 28 days) QTN (5, 10, or 20 mg/kg for 28 days)	LDH ↓ CK-MB ↓ AST ↓ ALT ↓ ALP ↓LDL-C ↓ VLDL-C ↓ MDA ↓ NO ↓ c-fos ↓ c-jun ↓ HDL-C ↑ SOD ↑ GSH↑ PPAR-γ ↑ Na-K-ATPase ↑ Complex I, II, IV ↑ Nrf2 ↑	[70]
Antioxidant enzyme cofactors	Zinc	Male Wistar rats; liver	NaAsO_2_ (100 ppm for 3 months) Zinc (227 mg/L for 3 months)	GSH ↑ SOD ↑ GPx ↑ GR ↑ CAT ↑ LPO ↓	[232]
	Selenium	SD rats; serum, liver	NaAsO_2_ (13 mg/L for 20 weeks) Na_2_SeO_3_ (17 mg/L for 20 weeks)	MDA ↓ Heat shock 70 kDa protein (HSP70) ↓ mRNA of *SOD1*, *CAT*, *GPx*, and *Thioredoxin Reductase 1* (*Txnrd1*) ↑	[233]
		PC12 cells	NaAsO_2_ (5, 10, 20, or 40 μM for 48 h) Na_2_SeO_3_ (5, 10, 20, or 40 μM for 48 h)	The cellular accumulation of arsenic ↓ mTOR/Akt autophagy signaling pathway ↑	[234]
